# Activate & fire: a feasibility study in combining acoustic stimulation and continuous theta burst stimulation in chronic tinnitus

**DOI:** 10.1186/s12883-022-03036-y

**Published:** 2023-01-12

**Authors:** Stefan Schoisswohl, Berthold Langguth, Franziska C. Weber, Mohamed A. Abdelnaim, Tobias Hebel, Martin Schecklmann

**Affiliations:** 1 Department of Psychiatry and Psychotherapy, University of Regensburg, Universitaetsstraße 84, 93053 Regensburg, Germany; 2 Department of Psychology, Universität der Bundeswehr München, Neubiberg, Germany

**Keywords:** Tinnitus, Theta burst stimulation, rTMS, Neuromodulation, Acoustic stimulation

## Abstract

**Background:**

Low frequency repetitive transcranial magnetic stimulation (rTMS) is commonly used to inhibit pathological hyperactivity of the auditory cortex in tinnitus. Novel and supposedly superior and faster inhibitory protocols such as continuous theta burst stimulation (cTBS) were examined as well, but so far there is not sufficient evidence for a treatment application in chronic tinnitus. rTMS effects in general are dependent on the brain state immediate before stimulation. This feasibility study was designed based on the concept to shift the pathological intrinsic brain state of tinnitus patients via acoustic stimulation (“activate”) and induce inhibitory effects via cTBS (“fire”).

**Methods:**

Seven tinnitus patients with response in residual inhibition received 10 consecutive daily sessions of a combinatory treatment comprised of 3-minute acoustic stimulation with white noise followed by 600 pulses of cTBS over the left temporo-parietal cortex (activate & fire). A control group of 5 patients was treated parallel to the activate & fire data collection with 10 sessions á 3000 pulses of 1 Hz rTMS over the left temporo-parietal cortex.

**Results:**

The activate & fire protocol was well tolerated except in one patient with tinnitus loudness increase. This patient was excluded from analyses. No statistical superiority of the activate & fire treatment approach in alleviating tinnitus-related symptoms was evident. Power calculations showed an effect size of 0.706 and a needed sample size of 66 for statistical significant group differences. On a descriptive level the activate & fire group demonstrated a stronger decrease in tinnitus-related symptoms.

**Conclusion:**

The present feasibility study showed that combining acoustic stimulation with magnetic brain stimulation may be well-tolerable in the majority of patients and represents a promising treatment approach for tinnitus by hypothetically alter the intrinsic state prior to brain stimulation.

## Introduction

Repetitive transcranial magnetic stimulation (rTMS) is a non-invasive brain stimulation method with the potential to induce cortical inhibition or excitation based on the applied protocol [1, 2]. Low-frequency stimulations (≤ 1 Hz) are deployed as inhibitory protocols and high-frequency stimulations as facilitatory protocols [1]. According to this heuristic, rTMS was used in a broad spectrum of neuro-psychiatric diseases [3] and was also applied and investigated as a potential treatment option for auditory phantom sounds such as acoustic hallucinations and tinnitus [4, 5]. Around 15% of the European population are affected by tinnitus, whereas 1–2% are severely concerned thereof [6]. Due to a wide variety of phenotypes and etiologies, there is so far no cure for tinnitus existent (cf. [7]). Dysfunctional neural changes, such as a disparity of neuronal inhibition and excitation along the auditory pathway provoking hyperactivity in auditory cortical regions [8, 9], provide a legit basis for the usage of inhibitory 1 Hz rTMS as a treatment option for tinnitus [10, 11]. However, the current state of research offers no unequivocal evidence for the effectiveness of 1 Hz rTMS in tinnitus – clinical trials demonstrate diverging findings as well as a high interindividual variability in treatment responses [12–15]. In addition to tonic rTMS other magnetic stimulation approaches were investigated such as theta burst stimulation (TBS). Thereby several bursts or trains of three pulses at 50 Hz are repeatedly delivered with an inter-burst-interval of 5 Hz (200 ms). TBS is considered a safe brain stimulation technique [16] and convinces with its short stimulation durations and its superiority in modulating cortical excitability compared to traditional rTMS protocols [17–19]. Via investigations of the healthy brain, it was shown that an intermittent application (iTBS) is capable to increase cortical excitation, whereas a continuous application (cTBS) decreases cortical excitation [20, 21].

A handful of studies investigated the consequences of inhibitory cTBS as a treatment approach for tinnitus showing an inhomogeneous picture with respect to its efficiency akin to 1 Hz rTMS [21–26]. For instance Chung et al. [27] report a greater alleviation of tinnitus severity by cTBS in contrast to a sham stimulation, whereas Schecklmann et al. [25] and Godbehere et al. [26] were not able to prove this superiority of cTBS. In a recent review about the usage of TBS in various neuro-psychiatric conditions it was concluded that there is currently no sufficient evidence for tinnitus [28]. This insufficient data basis is similar for cTBS treatment of acoustic hallucinations [5].

There is clear evidence that the effects of rTMS (and thus also TBS) are dependent on the intrinsic state of the brain immediate prior or during its administration [29–31]. By means of a sound stimulation before low frequency rTMS of the auditory cortex in healthy subjects, Weisz et al. demonstrated that the intended effects of inhibitory rTMS shifted respectively appeared to be state-dependent [32]. This might be of special interest for neuromodulation attempts in tinnitus. Since the intrinsic state of the tinnitus brain is already pathologically altered, it is obvious that neuromodulation effects might only partially appear as desired and that the supposed effects could even reverse and transfer from inhibitory to excitatory as indicated by many tinnitus patients not behaving according to the generally accepted heuristic of low-frequency-inhibitory and high-frequency-excitatory [1]. For instance, high-frequency protocols were shown to be effective in tinnitus as well [33, 34].

In tinnitus research it is commonly known that acoustic stimulations can induce short-term suppressions of the tinnitus percept. This phenomenon is termed residual inhibition and can be triggered in 60–80% of tinnitus cases [35–38]. Throughout this brief tinnitus suppression, it is assumed that a transient inversion of abnormal neural activity takes place [39, 40]. Hence, an acoustic stimulation in tinnitus patients before or during rTMS could theoretically shift the neural activity of tinnitus patients back to a “normal” state, which could facilitate heuristic-conform neuroplastic rTMS consequences (inhibitory = inhibitory; excitatory = excitatory).

With the present feasibility study we attempted to investigate this concept as a treatment approach in a small sample of tinnitus patients. By a stimulation with white noise (WN) we aimed to hypothetically alter the intrinsic state or rather briefly reverse tinnitus related brain activity (“activate”) in order to subsequently induce inhibitory effects by cTBS over the temporal cortex (“fire”). To draw conclusions about potential therapeutic advantages as well as the tolerability of this combination, treatment effects were contrasted against a control group treated in a parallel treatment with the regularly used rTMS protocol in a clinical context – 1 Hz rTMS over the temporal cortex.

## Materials and methods

All data were collected and analyzed within the framework of the Tinnitus Research Initiative database [41], which was approved by the ethics committee of the University of Regensburg, Germany (ethical approval number: 08/046).

### Sample characteristics

Patients with chronic tinnitus (> 6 months) were recruited from the Interdisciplinary Tinnitus Center Regensburg (Germany) in accordance with the following inclusion criteria: German-speaking; age between 18 and 75 years; no serious somatic, neurological or psychiatric conditions; non or stable psychotropic medication; no contraindications with respect to TMS; no current participation in any other tinnitus-related treatment or study. In addition, only patients experiencing residual inhibition were included (see section study procedure). Prior to study participation, each patient has been informed about objective, methods and potential side-effects and gave written informed consent.

7 patients (1 female) were found to be eligible and were included in the trial. Thereof one patient dropped out during the treatment phase due to an increase in tinnitus loudness. Thus, *N* = 6 patients (1 female) successfully finished the treatment phase and were considered for the present analyses.

### Study procedure

Over the course of the present trial 4 study visits were conducted consisting of a screening visit at any time prior to study start, a baseline visit at treatment start, an end of treatment visit directly after the intervention phase as well as a follow-up visit executed 12 weeks after treatment end. The intervention phase consisted of 10 treatment sessions of cTBS paired with acoustic stimulation (see section cTBS & acoustic stimulation) over 10 consecutive workdays (2 × 5 days; same daytime).

Patients had to fill out the German versions of the following tinnitus- and health-related questionnaires/assessments at all study visits: the Tinnitus Functional Index (TFI; [42]), the Tinnitus Handicap Inventory (THI; [43, 44]), the Major Depression Inventory (MDI; [45]) as well as Numeric Rating Scales (NRS) for tinnitus loudness (0—not at all loud; 10—extremely loud), for the discomfort caused by tinnitus (0—no discomfort; 10—severe discomfort), for tinnitus annoyance (0—not at all annoying; 10—extremely annoying), for tinnitus unpleasantness (0—not at all unpleasant; 10—extremely unpleasant) and for the ability to ignore the tinnitus perception (0—very easy to ignore; 10—impossible to ignore). In addition to the examination of patients’ study eligibility and the performance of informed consent procedures at screening, patients had to fill out the Tinnitus Sample Case History Questionnaire (TSCHQ; [46]), had to undergo standard clinical audiometric measurements (125 Hz–8 kHz; Madsen, Midimate, 622D, GN Otometrics, Taustrus, Denmark) and were tested for the induction of residual inhibition. Thereby a WN was presented diotically using conventional in-ear headphones (MDR-EX15LPB; Sony, Tokyo, Japan) together with an iPod Touch 7. Generation (Apple Inc., Cupertino, California, USA). The WN was created using Matlab (Matlab R2018b; Mathworks, USA), underwent a root-mean-square correction and featured a linear fade-in and fade-out phase of 1000 ms. Patients were instructed to increase the loudness of the WN until their tinnitus was successfully masked (starting from 35 dB SPL up to max. 85 dB SPL), which was followed by a three-minute WN stimulation and a subsequent evaluation if the tinnitus percept is diminished.

During end of treatment and follow-up visits, patients were additionally required to evaluate the improvement of their tinnitus-related complaints using the Clinical Global Impression Scale for Improvement (CGI-I; [47]) with a 7-point Likert Scale (1 = very much better; 2 = much better; 3 = minimally better; 4 = no change; 5 = minimally worse; 6 = much worse, and 7 = very much worse).

### Acoustic stimulation & cTBS (activate & fire)

Patients’ resting motor threshold (RMT) was determined prior to treatment onset via TMS single pulse administrations over the left primary motor cortex. RMT was defined as the minimum stimulation intensity needed in order to elicit motor evoked potentials (MEP) with a peak-to-peak amplitude of > 50 µV in not less than 50% of applied TMS pulses [2]. MEPs were recorded from the musculus abductor digiti minimi (ADM) of the right hand. Throughout the treatment phase all patients received ten sessions of cTBS á 600 pulses (110% RMT) applied over the left temporo-parietal cortex with the coil placed between the electrodes T3 and P3 as described in [48]. All magnetic stimulations were carried out using a MagPro X100 stimulator (MagVenture A/S, Farum, Denmark) together with an active-cooled figure-of-eight coil (Cool-B65 A/P; MagVenture A/S, Farum, Denmark).

On each treatment day the cTBS application was preceded by a three-minute acoustic stimulation with WN. Thereby the same WN stimulation procedure as already described above was applied. After each treatment session, patients had to rate the loudness of their tinnitus on a scale from 0 to 110% - whereas 0% signifies an absence of the tinnitus perception; 100% characterizes no change in the tinnitus percept and 110% represents a tinnitus loudness increase of 10%. If a tinnitus loudness suppression was present, the patients had to additionally rate the duration of the induced loudness decrease on the next day respectively before the start of the next treatment session.

### Control group − 1 Hz rTMS

To contrast the combination of WN and cTBS (activate & fire group) with a control group, data from the last tinnitus patients treated with the commonly used magnetic stimulation protocol for tinnitus at the Interdisciplinary Tinnitus Center Regensburg (Germany) was used. *N* = 5 tinnitus patients (1 female) were treated with 1 Hz rTMS over the left temporo-parietal cortex for 10 sessions á 3000 pulses with a stimulation intensity of 110% RMT. Data was equally collected under the framework of the Tinnitus Research Initiative database [41] (ethical approval number: 08/046) with an identical treatment schedule, procedure and patient eligibility criteria except susceptibility to RI.

### Statistical analysis

All statistical analysis were performed using the statistics software R (version 4.0.3; R Foundation for Statistical Computing, Vienna, Austria). The TFI was defined as the primary outcome measure for this feasibility study to analyze treatment effects as well as to conduct effect size and needed sample size calculations.

Differences between the activate & fire and the 1 Hz rTMS control group at screening were analyzed with two-sample t-tests for numerical and χ^2^-tests for categorical data. In case of a violation of parametric testing, Mann-Whitney-U-tests respectively Fisher’s exact tests were deployed. Data from study visits of both treatment groups were analyzed using linear mixed effect models separately for each assessment inventory (e.g., TFI). The model fitting followed the exact same procedure as already outlined in Schoisswohl et al., 2021 & 2022 [40, 49]. The following fixed effects as well as their interaction were tested in the model fitting procedure: time (screening, baseline, treatment end, follow-up), treatment group (activate & fire, 1 Hz rTMS). Potential differences within fixed effects were analyzed with post hoc tukey contrasts and adjusted for multiple comparisons using the tukey method. Effect sizes were evaluated with Cohen’s d.

Average score changes from baseline to treatment end (post – pre) were calculated for both groups and all assessment inventories and contrasted with two-sample t-test respectively Mann-Whitney-U-tests. Additionally, the corresponding effect size for these contrasts were calculated (Cohen’s d).

Potential differences between the treatment groups (activate & fire, 1 Hz rTMS) with respect to the CGI assessment (better, no change, worse) were evaluated separately for the treatment end and follow-up visit with χ^2^-tests or rather Fisher’s exact tests (cell frequency below 5).

Furthermore, the number of treatment responder was identified for the activate & fire as well as the 1 Hz rTMS control group by means of a 7-point reduction in the TFI [50] and a 7-point reduction in the THI [51]. Associations of treatment response (yes, no) with the respective treatment group (activate & fire, 1 Hz rTMS) were equally evaluated with χ^2^-tests respectively Fisher’s exact tests.

Pre to post treatment sum score changes were further used to derive a sample size estimation with G*Power [52] in favor of the group contrast – activate & fire vs. 1 Hz rTMS (5% alpha level; 80% statistical power; two-sided) for our primary outcome the TFI as well as all other assessment inventories.

Mean tinnitus loudness suppression after treatment sessions for the activate & fire group and its potential duration are reported descriptively.

## Results

### Sample characteristics, group differences, side effects

The tinnitus sample (*n* = 6) at hand had a mean age of 49.17 years (SD = 16.12) and indicated an average tinnitus duration of 96.20 months (SD = 126.35). Tinnitus severity, as measured by the TFI and THI, manifested in moderate to major severity (TFI: M = 57.67, SD = 10.66; THI: M = 49.00, SD = 16.04). No depressive disorder was evident at the screening visit (MDI: M = 12.67, SD = 3.83). The average RMT (%) for magnetic stimulations was 46.17 (SD = 12.04).

Statistical contrasts of descriptive data between the activate & fire and the 1 Hz rTMS control group as listed in Table 1 revealed no significant differences at screening.

Besides the above-mentioned drop-out due to tinnitus loudness increase, no further TMS-related side effects were reported in the activate & fire group. In the 1 Hz rTMS group three patients experienced side effects: tinnitus loudness increase (1 patient), slight vertigo (1 patient) and hypertonia together with headache (1 patient), but none of them dropped out.

Average loudness of the WN acoustic stimulation in the activate & fire group was 64.60 dB SPL (SD = 13.19, Min = 44.55, Max = 81.97).

**Table 1:** Sample characteristics

	Activate & Fire				1 Hz rTMS					
N (female)	6 (1)				5 (1)					
	**M ± SD**	**Md**	**Min**	**Max**	**M ± SD**	**Md**	**Min**	**Max**	**T**_**(df)**_**/ U**	**p**
Age (years)	49.17 ± 16.12	53.50	27.00	66.00	52.40 ± 12.05	48.00	37.00	67.00	- 0.38 _(8.94)_	0.713
Tinnitus duration (months) (1/2 missing)	96.20 ± 126.35	39.00	34.00	322.00	219.14 ± 137.98	257.00	66.00	334.00	2.00	0.143
Hearing loss left (dB) (1/1missing)	10.73 ± 9.58	5.71	1.83	21.67	17.22 ± 9.15	18.89	6.11	25.00	-1.04 _(6.70)_	0.336
Hearing loss right (dB) (1/1missing)	9.84 ± 9.65	5.00	1.83	23.33	10.28 ± 6.77	9.44	3.89	18.33	- 0.08 _(6.94)_	0.938
RMT (%)	46.17 ± 12.04	47.00	30.00	60.00	38.00 ± 8.15	36.00	30.00	51.00	1.33 _(8.72)_	0.216
TFI score (0-100) (0/1missing)	57.67 ± 10.66	54.00	46.40	77.20	48.05 ± 26.85	50.50	16.80	74.40	0.68 _(3.64)_	0.537
THI score (0-100) (0/1missing)	49.00 ± 16.04	44.00	36.00	78.00	41.00 ± 16.85	40.00	24.00	60.00	0.75 _(6.33)_	0.480
MDI score (0-50) (0/1missing)	12.67 ± 3.83	11.50	9.00	20.00	10.75 ± 6.70	11.00	4.00	17.00	0.52 _(4.33)_	0.630
NRS tinnitus loudness (0-10) (0/1missing)	6.50 ± 2.07	6.50	4.00	10.00	6.50 ± 1.91	7.00	4.00	8.00	0.00 _(6.97)_	≥ 0.999
NRS tinnitus discomfort (0-10) (0/1missing)	8.57 ± 1.37	9.00	7.00	10.00	7.75 ± 2.22	8.00	5.00	10.00	0.74 _(4.54)_	0.497
NRS tinnitus annoyance (0-10) (0/1missing)	7.67 ± 2.73	8.50	4.00	10.00	7.50 ± 1.29	7.50	6.00	9.00	0.13 _(7.51)_	0.901
NRS tinnitus ignorability (0-10) (0/1missing)	7.50 ± 1.64	7.50	5.00	10.00	8.75 ± 0.96	8.50	8.00	10.00	-1.52 _(7.95)_	0.168
NRS tinnitus unpleasantness (0-10) (0/1missing)	7.00 ± 2.00	7.00	4.00	10.00	8.00 ± 1.83	8.00	6.00	10.00	- 0.82 _(7.02)_	0.441

*RMT*  Resting Motor Threshold, *TFI*  Tinnitus Functional Index, *THI *Tinnitus Handicap Inventory, *MDI*  Major Depression Inventory, *NRS *Numeric Rating Scale

### Treatment results

Linear mixed effect model fitting revealed the model response ~ time + (1 | patient id) with a significant effect of time for the THI as well as the NRSs for tinnitus annoyance and ignorability. For the MDI the model response ~ time + treatment group + time*treatment group + (1 | patient id) could be identified. Fixed effect testing revealed a significant interaction of time*treatment group.

**Fig. 1:**
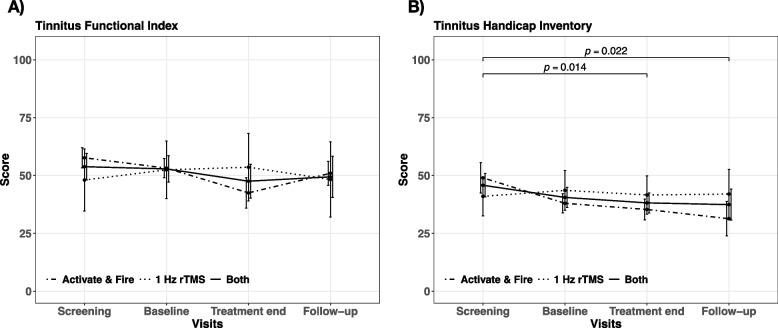
Time course and results of post hoc analysis. Total score changes for all study visits (screening, baseline, treatment end, follow-up) are illustrated for the (**A**) Tinnitus Functional Index, (**B**) Tinnitus Handicap Inventory and separately for the activate & fire group (dot-dashed line), the 1 Hz rTMS control group (dotted line) as well as the average of both groups (solid line). Error bars indicate standard errors. Significant differences are highlighted with bars and the corresponding *p*-values

**Fig. 2:**
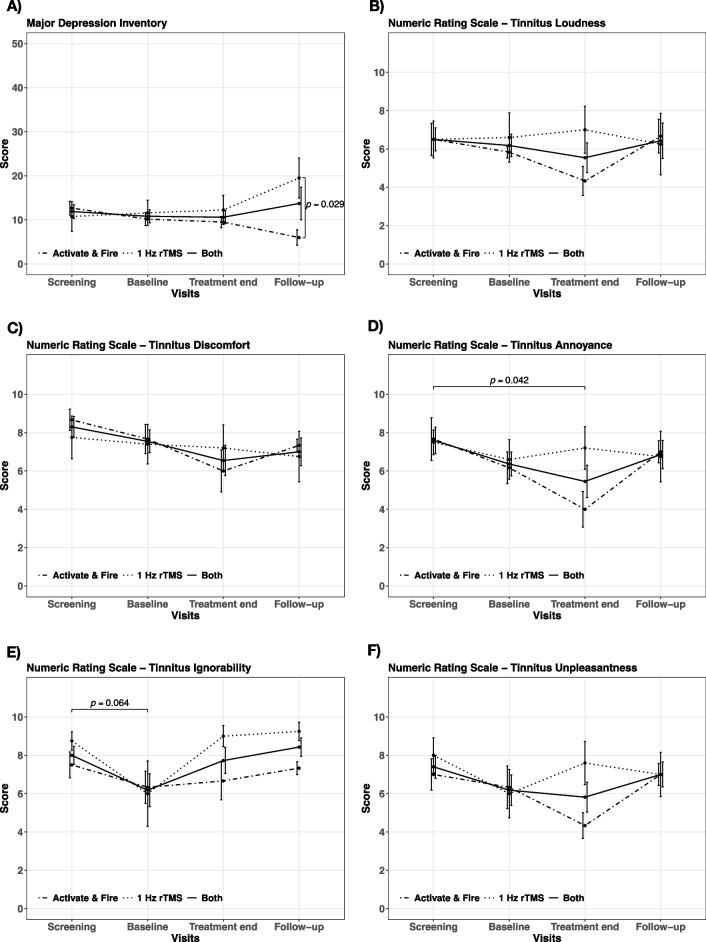
Time course and results of post hoc analysis. Total score changes for all study visits (screening, baseline, treatment end, follow-up) separately for the activate & fire group (dot-dashed line), the 1 Hz rTMS control group (dotted line) and the average of both groups (solid line) are illustrated for the (**A**) Major Depression Inventory and the Numeric Rating Scales for (**B**) tinnitus loudness, (**C**) tinnitus discomfort, (**D**) tinnitus annoyance, (**E**) tinnitus ignorability and (**F**) tinnitus unpleasantness. Error bars indicate standard errors. Significant differences are highlighted with bars and the corresponding *p*-values

Ensuing post hoc tukey contrasts showed significant differences between treatment end and screening for the THI (Fig. 1B) and the NRS for tinnitus annoyance (Fig. 2D), whereby the THI score and tinnitus annoyance decreased from screening to treatment end irrespective of treatment group. Further the THI score appeared to be significant lower at follow-up in contrast to screening (Fig. 1B). A statistical trend towards a reduction from screening to baseline could be observed for the NRS tinnitus ignorability (Fig. 2E). No statistically significant differences between baseline, treatment end or follow-up were evident for any of the assessment inventories, neither for the individual treatment groups nor in general.

Post hoc contrasts for the MDI revealed a significant difference between the activate & fire and 1 Hz rTMS group exclusively at follow-up (Fig. 2 A). Detailed results of post hoc contrasts showing statistically relevant differences can be seen from Table 2. Mean scores per treatment group at each study visit are illustrated in Figs. 1 and 2 for each assessment inventory.

**Table 2:** Significant post-hoc tukey contrasts

Contrast	M ± SD	Estimate	T _(df, se)_	*p*	d
**THI – both groups**					
Treatment end – screening	38.18 ± 14.35 – 45.80 ± 15.96	-9.54	-3.25 _(31.40, 2.93)_	0.014	0.502
Follow-up – screening	37.43 ± 17.73 – 45.80 ± 15.96	-10.55	-3.06 _(31.80, 3.44)_	0.022	0.496
**MDI**					
Follow-up:					
Activate & Fire vs. 1 Hz rTMS	6.00 ± 3.00 – 19.50 ± 9.11	-13.01	-3.42 _(38.20, 3.81)_	0.029	1.991
**NRS – Tinnitus annoyance**					
Treatment end – screening	5.45 ± 2.81 – 7.60 ± 2.17	-2.32	-2.79 _(31.70, 0.83)_	0.042	0.856
**NRS – Tinnitus ignorability**					
Baseline – screening	6.18 ± 2.82 – 8.00 ± 1.49	-1.95	-2.60 _(31.60, 0.75)_	0.064	0.807

*RMT* Resting Motor Threshold, *TFI* Tinnitus Functional Index, *THI* Tinnitus Handicap Inventory, *MDI* Major Depression Inventory, *NRS* Numeric Rating Scale

No significant difference between the activate & fire and 1 Hz control group regarding pre to post treatment score changes could be observed. Only a trend towards significance was present for the NRS loudness and unpleasantness with the activate & fire group featuring a higher score decrease.

On a descriptive level the activate & fire group exhibited not only decreases in almost all assessments from pre to post treatment but also a stronger reduction compared to the control group. Except for the NRS tinnitus ignorability which slightly increased (remark: more difficulties to ignore tinnitus), though deterioration was still less than for the control group. Average score changes from pre to post treatment plus results of statistical contrast are summarized in Table 3.

**Table 3:** Pre to post average score changes per treatment group, statistical contrast and sample size estimation

	Activate & Fire	1 Hz rTMS				Sample size
	M ± SD	M ± SD	T _(df)_ / U	p	d	
**TFI**	-10.69 ± 21.68	1.12 ± 6.43	9.00	0.329	0.706	66
**THI**	-2.67 ± 9.27	-2.00 ± 8.48	-0.12 _(8.88)_	0.904	0.075	5584
**MDI**	-0.67 ± 5.46	0.65 ± 3.10	-0.15 _(7.92)_	0.882	0.289	378
**NRS – Tinnitus loudness**	-1.50 ± 2.07	0.40 ± 0.55	5.50	0.088	1.198	24
**NRS – Tinnitus discomfort**	-1.67 ± 4.27	-0.20 ± 0.45	15.00	> 0.999	0.460	152
**NRS – Tinnitus annoyance**	-2.16 ± 3.49	0.60 ± 1.95	7.00	0.160	0.949	38
**NRS – Tinnitus ignorability**	0.33 ± 2.34	3.00 ± 3.87	7.50	0.187	0.857	46
**NRS – Tinnitus unpleasantness**	-2.00 ± 4.05	1.60 ± 2.07	-1.90 _(7.69)_	0.096	1.085	30

*TFI*  Tinnitus Functional Index, *THI*  Tinnitus Handicap Inventory, *MDI *Major Depression Inventory, *NRS *Numeric Rating Scale, *d *Cohen´s d. Negative values indicate a decrease in mean scores from pre to post treatment

A Fisher’s exact test found no statistically significant association of patients’ CGI ratings (better, no change, worse) with treatment group (activate & fire, 1 Hz rTMS) neither at treatment end nor at follow-up.

By means of predetermined responder criteria, we can report an overall quantity *n* = 3 (27.27%) treatment responder in our primary outcome measure the TFI, whereas two responder (33.33%) were identified in the activate & fire group and one responder (20%) was identified in the 1 Hz rTMS treatment group. Using the THI, a total number of 4 treatment responder (36.36%) could be identified. Three of which belong to the activate & fire group (50%) and one to the 1 Hz rTMS group (20%). One patient in the activate & fire group appeared to be a treatment responder when response was defined by score changes in both the TFI and THI. No significant association of TFI or THI treatment responder (yes/ no) with treatment group (activate & fire, 1 Hz rTMS) was evident.

With an effect size of d = 0.706, derived from mean score changes from baseline to treatment end in our primary outcome measure the TFI for the activate & fire and the 1 Hz rTMS control group, a sample size of *N* = 66 would be necessary for a proper comparison of these two groups. Effect sizes and sample size estimations vary greatly between the used assessment inventories (cf. Table 3**)**.

Additional tinnitus loudness ratings after each session in the activate & fire group exposed tinnitus suppressions in 5 out of 6 patients (83.33% patients). The average loudness rating after WN followed by cTBS was 88.56% (SD = 13.20, Min = 50.00, Max = 110; 0% - absence of tinnitus percept, 100% - no change, 110% - 10% tinnitus loudness increase). Tinnitus suppression persisted from three minutes up to a few hours.

## Discussion

The purpose of the current pilot study was to evaluate the feasibility and tolerability of a combinatory treatment of acoustic stimulation paired with magnetic brain stimulation in small sample of tinnitus patients. Through a WN stimulation prior to cTBS applied over the temporo-parietal cortex we intended to induce short-term acoustic tinnitus suppression (RI) accompanied by a temporal inversion of tinnitus-associated brain activity [39, 40] and thus modulate the intrinsic brain state. As past studies suggested state-dependency of rTMS [32], we hypothesized that such a combination is supposed to induce positive treatment effects and contrasted this with the commonly used neuromodulation treatment of 1 Hz rTMS.

With the present feasibility study, we are not able to demonstrate a statistical superiority of such a combination (activate & fire) in alleviating tinnitus distress in contrast to a control group (1 Hz rTMS). The concept of bimodal auditory and central stimulation has already been investigated by combinations of acoustic stimulation/ sound therapies with transcranial direct current stimulation, likewise demonstrating inconclusive findings so far [53–55]. However, acoustic stimulation combined with stimulation of cranial nerves such as the vagus or the trigeminal nerve shows promising findings [56–59].

Nevertheless, we can report an amelioration of tinnitus-related symptoms for the activate & fire group plus a superior alleviation in contrast to the 1 Hz rTMS control group on a descriptive level (cf. Figures 1 and 2; Table 3). Further the quantity of treatment responder appeared to be higher for the activate & fire group (TFI: 33.33% vs. 20%; THI: 50% vs. 20%). These findings should not be overinterpreted, since we could not observe an association of treatment group neither with treatment response nor clinical improvement at treatment end or follow-up.

The combination of acoustic and magnetic stimulation appeared to be well-tolerated and featured less reported side-effects than the control group (tinnitus loudness increase, slight vertigo, hypertonia together with headache). The only observed side effect was a tinnitus loudness increase in one patient which led to treatment discontinuation, even though we screened for the possibility to induce residual inhibition prior study participation. Tinnitus loudness increases respectively residual excitation are not uncommon after sound stimulation [60]. However, as the patient experienced short-term acoustic tinnitus suppression during screening and residual inhibition is assumed to be a reliable phenomenon [61–63], the reason for an increase might lie in the combination of both interventions.

It could be possible that in this specific patient RI did not trigger a temporal shift of pathological brain activity and thus inhibitory effects did not take place as intended. Similar to one patient reporting a tinnitus loudness increase in the control group.

One unanticipated finding was the significant difference in depressive symptoms at follow-up. While MDI scores increased in the control group, sum scores decreased in the activate & fire group and appeared to be significant lower at follow-up. Depression represents a relative common comorbidity in tinnitus [64–66]. As prefrontal cortex stimulation is an established treatment for depression [3], past studies aimed for targeting this region in order to reduce depressive symptoms in tinnitus [48, 67–69]. In the present study we solely targeted the temporal cortex. Therefore, a potential explanation for the available reduction in depressive symptoms might be that descriptive but yet considerable ameliorations in tinnitus-related symptoms (baseline to treatment end) observed for the activate & fire group provoke a decrease in depressive symptoms on a long-term perspective (follow-up). However, this result should be interpreted with great caution, as the data comes from a small sample and requires confirmation in a larger sample, before further conclusion can be drawn.

In favor of an adequate statistical comparison of these two groups (activate & fire vs. 1 Hz rTMS control group) using the TFI, a sample of *N* = 66 tinnitus patients would be necessary. As can be seen from Table 3, sample size calculations greatly differ between the used assessment inventories. While group contrasts based on the NRS for tinnitus loudness (d = 1.198, *N* = 24) and unpleasantness (d = 1.085, *N* = 30) show large effect sizes, and therefore also the need of rather small samples, an estimation based on the THI demonstrated very small effect sizes together with a very large (unfeasible) required sample size (d = 0.075, *N* = 5584) for a prober group contrast.

It was stated that the THI might be more suitable to capture the psychological dimensions of tinnitus distress and should be primarily used in the context of psychological-based treatments [70], hence the THI does not appear to be fully appropriate to track changes in tinnitus distress after neuromodulation interventions. However, we investigated a relatively small sample of tinnitus patients in the present feasibility study, therefore our sample size estimation should be interpreted with caution. Further pilot studies investigating a somewhat larger tinnitus sample are needed for more detailed power and sample size calculations.

With respect to general treatment efficacy, we could not demonstrate a therapeutic effect of rTMS akin to past studies [4, 14]. This is potentially driven by the given divergence between the two groups in tinnitus-related assessments - amelioration in the activate & fire group and deterioration in the control group - resulting in a disappearance of therapeutic effects on average (cf. Figures 1 and 2).

Even though the present findings are hampered by a lack of statistical superiority, which is very probable due to the small sample size (feasibility study), our results are in favor of the activate & fire approach and therefore suggests further investigations in this regard.

Future studies should aim for in-depth and systematic investigations by means of an appropriate sample size. Furthermore, the relevance of the temporal relationship between acoustic stimulation and magnetic stimulation remains to be explored. Here we investigated the application of the cTBS series after offset of WN. An alternative option would be the application of cTBS simultaneously with auditory stimulation. Moreover, modification of the parameters of both auditory stimulation and TMS may impact the efficacy, resulting in a large potential parameter space. For the exploration of this large parameter space small pilot studies seem a feasible option, as they can reveal potential “candidate parameters” and provide the necessary knowledge for designing sufficiently powered larger confirmatory studies.

Although we could observe tinnitus loudness reductions after WN stimulation in the activate & fire group in 5 out of 6 patients, future research should strive for a systematic assessment of potential short-term loudness decreases or should even consider to start with brain stimulation once a patient’s tinnitus percept is suppressed. Further, different sound stimuli should be tested per patient in regards to RI induction as past research suggests that different tinnitus subtypes respond differently to certain stimuli [38].

Besides the advantages of TBS in shorter stimulation durations which allowed for a safe and fast treatment administration in accordance with the local hospital requirements during the COVID-19 pandemic, burst stimulation is assumed to produce stronger effects in tinnitus suppression through stimulating neurons from both the extra- and lemniscal system, whereas tonic rTMS only modulates the lemniscal system [71, 72]. As TBS might already be superior on its own, future studies should strive for a direct contrast of burst stimulation protocols either with or without combined acoustic stimulation on top of a comparison with the clinically commonly used 1 Hz rTMS protocol, in order to draw clear conclusions about the consequences of such a combinatory treatment.

## Conclusion

The present feasibility study evaluated if a novel combination of acoustic and magnetic stimulation is superior to the traditionally used tinnitus treatment approach of 1 Hz rTMS. As past studies suggested state-dependency of rTMS, we attempted to modulate the intrinsic brain state with a WN application prior to cTBS and therefore improve treatment efficacy. The combination of WN and cTBS appeared to be well tolerated and featured less side effects than 1 Hz rTMS, which endorses more refined examinations of such a combination in future studies. Despite the lack of a statistical superiority due to small sample size, the present findings indicate a stronger descriptive alleviation of tinnitus-related symptoms in support of this combinatory approach. Additionally, depressive symptoms were significantly less at follow-up for the combinatory treatment group. Further in-depth and systematic research by means of an appropriate sample size would be worthwhile as the present results are supportive of a combinatory treatment of acoustic and magnetic brain stimulation for tinnitus.

## Data Availability

The datasets used and/or analysed during the current study are available from the corresponding author on reasonable request.
